# Randomized Trial Demonstrating No Translocation of Intact Intestinal Bacteria During Hemodialysis or Hemodiafiltration

**DOI:** 10.1016/j.ekir.2024.09.025

**Published:** 2024-10-10

**Authors:** Paul A. Rootjes, Muriel P.C. Grooteman, Andries E. Budding, Hetty J. Bontkes, Gertrude Wijngaarden, Menso J. Nubé, Camiel L.M. de Roij van Zuijdewijn

**Affiliations:** 1 Amsterdam UMC, location AMC, Nephrology, Amsterdam, the Netherlands; 2 Amsterdam Cardiovascular Sciences (ACS), Diabetes and Metabolism, Amsterdam, the Netherlands; 3 Department of Internal Medicine, Gelre Hospitals, location Apeldoorn, Apeldoorn, the Netherlands; 4 InBiome, Amsterdam, the Netherlands; 5 Department of Clinical Chemistry, Medical Immunology Laboratory and Amsterdam Infection and Immunity, Amsterdam UMC, Amsterdam, the Netherlands; 6 Department of Internal Medicine, Spaarne Gasthuis, Haarlem, the Netherlands

**Keywords:** hemodiafiltration, hemodialysis, bacterial DNA, intestinal translocation, acute phase response (APR), randomized cross-over trial

## Abstract

**Introduction:**

The low incidence of intradialytic hypotension (IDH) in high-volume (HV) hemodiafiltration (HDF) may help in maintaining gut perfusion during treatment. Preservation of gut endothelial integrity would limit or prevent bacterial translocation and subsequent systemic inflammation, which may contribute to the low mortality rate in HV-HDF.

**Methods:**

Forty patients were cross-over randomized to standard (hemodialysis [HD]) (S-HD), cool HD (C-HD), and HDF (low-volume [LV] and HV, convection volume (CV) of 15 L and ≥ 23 L per session, respectively), each for 2 weeks. Quantitative assessment of microbial DNA (mDNA) in blood was performed before and after dialysis by 16S to 23S interspace profiling after DNA isolation. The intradialytic acute phase response (APR) was evaluated by high-sensitivity C-reactive protein (hs-CRP), interleukin-6 receptor (IL-6R), soluble CD14 (sCD14), and vascular-cell-adhesion molecule-1 (VCAM-1). Differences between modalities were primary objectives.

**Results:**

mDNA was absent from all samples. IL-6R, sCD14, and VCAM-1 increased equally in all modalities (median increase: 12.5%, 14.0%, 14.8%, respectively; *P* < 0.05). hs-CRP increased only in C-HD and HV-HDF (median increase: 12.6%, *P* < 0.05). After correction for hemoconcentration, most APR markers decreased (median: sCD14, −11.3% and VCAM-1, −14.4% in all modalities; IL-6R, −13.4% in C-HD, LV-HDF, and HV-HDF; *P* < 0.05). hs-CRP only decreased in C-HD (−13.5%, *P* = 0.004).

**Conclusion:**

From this study, we conclude as follows: (i) circulating mDNA could not be demonstrated; (ii) in the crude analysis, a similar APR was noted in all modalities, individual markers remained stable or declined after correction for hemoconcentration; and (iii) because neither bacterial translocation nor an APR was observed in either modality, it is highly unlikely that the superior survival of HV-HDF is explained by a superior preservation of gut integrity.

## Graphical abstract

**Figure:**
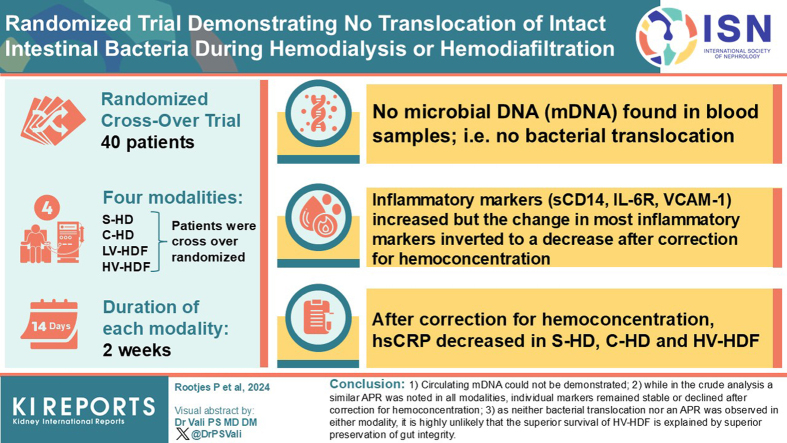

See Commentary on Page 12

Despite substantial medical and technological progress over the years, the mortality rate of patients with end-stage kidney disease remains high. In this respect, both patient-related, disease-specific, and treatment-associated factors appear to be involved. Even though HD is a life-prolonging treatment, its application is hampered by side-effects, both during the treatment and in the long term. In the short-term, both patient-reported discomfort and physical complications, such as IDH and bio-incompatibility issues,^1^, ^2^, ^3^ are frequently observed. Notorious illustrations of long-term adverse sequelae of intermittent HD are recurrent access problems, low-grade inflammation, and persistent fluid overload.^4^

As indicated by 2 meta-analyses^5^^,^^6^ and recently confirmed in a large randomized controlled trial,^7^ survival of patients with end-stage kidney disease is substantially prolonged by online post-dilution HV (CV ≥23 L/treatment) HDF, if compared to high-flux HD. However, it is unclear why HV-HDF improves survival. Neither the superior removal of middle molecular weight (MW) uremic toxins,^8^ nor long-term reductions in inflammation and oxidative stress have convincingly been associated with a lower mortality.^9^ Therefore, other factors such as a low IDH incidence and preserved organ perfusion,^10^, ^11^, ^12^, ^13^ may underlie its beneficial effect on survival.

Both a reduced circulating blood volume, caused by obligate ultrafiltration (UF), a compromised peripheral vascular resistance and an inadequate plasma refill rate^14^ may contribute to IDH, which in turn has been linked to reduced organ perfusion, tissue injury, and mortality.^15^ However, definite proof that IDH provokes organ damage and that its prevention reduces tissue injury is absent. Intradialytic perfusion deficits, whatever their causes, have not only been observed in the heart,^16^ but also in skin, brain,^17^ kidneys,^18^ and hepato-splanchnic region.^19^ Because microcirculatory dysfunction is common in patients with end-stage kidney disease,^20^ additional systemic circulatory stress may further compromise the functioning of vital organs, including the gut. As observed in unstable patients in the intensive care unit,^21^ disruption of the intestinal epithelial barrier may permit the transfer of intact mDNA to the circulation.^22^ Similar alterations may occur in fragile patients on HD, who often suffer from fluid overload, slow intestinal transit time, and impaired protein assimilation.^23^^,^^24^ Together with medication that interferes with gut acidity, these abnormalities may promote bacterial dysbiosis, reduced gut epithelial barrier function, and bacterial translocation.^23^

IDH occurs less often during HV-HDF than during S-HD^13^ and previous research indicated that chronic microinflammation is lower in patients on HDF.^9^^,^^25^ Because IDH may cause a reduction in tissue blood flow in patients with a compromised microcirculation, we speculated that both the intradialytic translocation of intact bacteria from the bowel to the blood and the resulting APR would be less manifest in HV-HDF than in HD. Therefore, we assessed the appearance of mDNA in the circulation and the generation of an APR during 4 extracorporeal replacement therapies and attempted to evaluate their correlations with IDH.

## Methods

### Study Design

The current investigation is part of an open-label, cross-over, multicenter randomized controlled trial in prevalent dialysis patients (ClinicalTrials.gov identifier NCT03249532). Methods have been described elsewhere.^26^ Briefly, patients were randomized to S-HD (with high-flux membrane, dialysate temperature of 36.5 ^o^C), C-HD (dialysate temperature of 35.5 ^o^C), LV-HDF (dialysate temperature of 36.5 ^o^C, target CV 15 L per session), and HV-HDF (dialysate temperature of 36.5^o^C, target CV ≥23 L per session), all during 2 weeks. The study lasted 10 weeks in total, was conducted in accordance with the Declaration of Helsinki and Good Clinical Practice guidelines and approved by the Medical Ethical committee (METc) of VU University medical center (METc VUmc: 2017.581/NL61210.029.17). Written informed consent was obtained from all patients.

### Study Population

From July 2018 to February 2021, 45 patients were recruited from 3 centers in the Netherlands: Niercentrum aan de Amstel, Amstelveen; Amsterdam UMC, location VU University medical center, Amsterdam; Sint Antonius hospital, Nieuwegein. Inclusion criteria were the following: (i) treatment with HD(F) 3 times/week for 4 hours for ≥2 months, (ii) ability to understand the study procedures, (iii) willingness to provide informed consent, (iv) dialysis single-pool Kt/V_urea_ ≥1.2, (v) blood flow rate feasibility ≥350 ml/min, and (vi) access recirculation <10%. Exclusion criteria were the following: age < 18 years, life expectancy < 3 months, participation in another clinical intervention trial, and/or severe noncompliance.

### Dialysis Prescription and Equipment

Dialysis characteristics are described elsewhere.^26^ In short, treatments were performed with Xevonta 23 high-flux dialyzers (B. Braun Avitum AG, Melsungen, Germany) on Dialog iQ machines, including the captive lines Diastream (both B. Braun Avitum AG, Melsungen, Germany). Treatment time was fixed at 4 hours per session. In all 3 dialysis centers, ultrapure dialysis fluids were prepared online with a comparable quality (< 0.1 colony forming units/ml and < 0.03 endotoxin units/ml) and subjected to regular quality check (weekly assessment of bacterial growth and endotoxin content) and mixed using Sol-Cart Bicarbonate cartridge and acidic dialysate. Substitution fluid was prepared by 1 additional UF-step (Diacap Ultra, B. Braun Avitum AG, Melsungen, Germany), before infusing into the blood. Apart from the modality, settings and devices were kept unaltered. Patient care was performed according to national and international quality of care guidelines.^27^^,^^28^

### Primary and Secondary End Points

Primary objectives of this analysis were potential differences between modalities in the change of circulating mDNA and the APR during a single dialysis session. The secondary end points were correlations between mDNA/APR and IDH. IDH was defined as a systolic blood pressure < 90 mm Hg or < 100 mm Hg, providing a predialysis systolic blood pressure < 160 mm Hg or ≥ 160 mm Hg respectively, independent of symptoms or interventions.^29^

### Data Collection

#### Baseline Data Registration

At baseline, information on demographics, comorbidity, primary renal diagnosis, and medication was noted. Body weight and interdialytic weight gain was assessed before dialysis. Data on vascular access, access flow, anticoagulation, needle size and type, blood-pump speed, dialysis machine, and dialyzer were documented as well. CV, calculated as the sum of intradialytic weight loss and substitution volume (L/session), was noted in all HDF sessions.

#### Hemodynamic Monitoring

During the last 3 sessions of each modality, blood pressure was recorded before treatment and every 15 minutes thereafter, using an automated manometric cuff-device connected to the dialysis machine (Dialog iQ, automatic blood pressure monitor, B. Braun Avitum AG, Melsungen).

#### Blood Sampling

All blood samples were drawn after the long interdialytic interval in the sixth (last) session of each modality from the arterial line of the extracorporeal circuit with a 21-gauge needle before the actual start of treatment (but after administration of low MW heparin) and after 4 hours of treatment. The blood flow rate at the moment of blood sampling was 350 to 400 ml/min. For each patient, treatment settings were kept similar during all 4 treatment modalities.

#### mDNA

To maximize mDNA recovery, extraction was performed with the Polaris method, as described before.^30^ In short, 5.0 ml of blood was mixed with an equal volume of selective buffer for 3 minutes, to lyse blood cells and fragment released human DNA, after which 5.0 ml neutralization buffer was added. The selective lysis degrades human cell membranes but not bacterial cell walls. Considering that an elevated pH ensures degradation of released nucleic acids, this method focuses on enrichment of intact bacteria. Given that gram-negative bacteria might be lysed upon prolonged exposure, an equal volume of neutralization buffer was added, ensuring complete arrest of the selective lysis by lowering pH and diluting the detergent to ineffective concentrations. Up to this point, bacteria remain intact. Consecutively, suspensions were centrifuged for 15 minutes at 2791 g and the pellets were resuspended in 1.0 ml washing buffer and centrifuged for 10 minutes at maximum speed in an Eppendorf centrifuge. The resulting pellets were thoroughly resuspended in 200 μl bacterial lysis buffer and incubated for 10 minutes at 95 °C on a thermomixer set at 1000 rpm. After addition of 20 μl neutralization buffer 2, lysates were further processed for DNA purification using the generic program of the EasyMAG device (BioMerieux, Marcy L’Etoile, France). Detection and identification of mDNA was done with the Molecular Culture assay (inBiome, Amsterdam, the Netherlands), a method optimized for highly sensitive detection of mDNA, based on the IS-pro technique.^31^^,^^32^ IS-pro is an eubacterial polymerase chain reaction-based technique for detection of all bacterial species within a sample, based on length and sequence polymorphisms of bacterial 16S-23S ribosomal interspace regions. Because these regions are present in all bacteria and their polymorphisms are highly species-specific, IS-pro is a universal system that can detect and identify the vast majority of bacterial species that colonize or invade the human body. Given that the technique has been set up to minimize interspecies polymerase chain reaction competition, IS-pro can simultaneously detect and identify many species, rendering the technique applicable to single-species detection and complex microbiota analysis alike. The combination of the molecular culture assay with the Polaris extraction has been optimized to detect bacterial loads as low as 1 CFU per 5.0 ml blood. A positive control was included for each polymerase chain reaction run of 96 samples and a negative control for each DNA isolation batch of 24 samples. Because we used a high-sensitive, state-of-the-art polymerase chain reaction-based assay to measure mDNA, which has only been applied so far in septic patients, for logistical and ethical reasons, we executed sequential hypothesis testing by performing an interim analysis on the samples of the first 11 patients.

#### Inflammation Markers

Given that small substances may pass high-flux membranes, only larger APR markers were measured in all patients (*N* = 40). Apart from the specific inflammation markers, sCD14 (MW 54 kilo Dalton), IL-6R (MW 50 kilo Dalton) and hs-CRP (MW 120 kilo Dalton), VCAM-1 (MW 110 kilo Dalton) were assessed as indicators of inflammation-induced endothelial injury.^33^ EDTA and heparin samples were centrifuged at 1800 g for 10 minutes within 30 minutes after withdrawal and thereafter placed on ice and stored at −80 ^o^C until assessment. Accordingly, markers were determined by ELISA (R&D Systems, Minneapolis, MN) and by particle-enhanced immunoturbidimetric assay (Roche Cobas Chemistry Analyzer, Roche Diagnostics GmbH, Mannheim, Germany), respectively, according to the manufacturer’s instructions.

### Statistical Methods

Baseline characteristics were summarized as mean (SD) for normally distributed continuous variables, median (interquartile range) for non-Gaussian distributed continuous variables, and counts (percentages) for categorical variables. Potential differences in the rate of change over a dialysis session in concentrations of mDNA, sCD14, IL-6R, VCAM-1, and hs-CRP between dialysis modalities, various subcategories of IDH susceptibility and between sessions with and without IDH were evaluated. In all cases, model assumptions were checked and not violated. For all linear mixed effect models, the best model fit was determined by the lowest Aikaike’s Information Criterion and included a random intercept, a random slope or both. A 2-sided *P* ≤ 0.05 was considered statistically significant. Statistical evaluations were performed with statistical software package SPSS version 26.0 (IBM Inc., IL).

#### Differences Between Modalities

First, we visualized changes of mDNA, sCD14, IL-6R, VCAM-1, and hs-CRP as stratified by modality. Because mDNA was not found in any of the samples of the first 11 patients, further analysis of this marker was not performed. Next, we evaluated whether the predialytic values of all inflammation markers were similar in the 4 modalities using a repeated measures analysis of variance after log-transformation, given their nonparametric distribution. If the assumption of sphericity as determined by Maucly’s test was violated, we used Greenhouse-Geisser correction. To assess potential differences in the rate of change between modalities per inflammation marker, linear mixed effect models were fitted with an interaction term between modality and time. Next, stratified linear mixed effect models were used to determine the rate of change of every APR marker per modality.

#### Correction for Hemoconcentration

In separate analyses, the influence of hemoconcentration on the changes of each individual APR marker was evaluated. Postdialytic values were corrected for UF volume. To this end, we first calculated adjusted postdialytic APR values using the serum hematocrit (Ht) in available patients (*n* = 11) with the formula:^34^ corrected postdialytic APR marker_tx_ = crude postdialytic APR marker_tx_ × (Ht_t0_/[1 − Ht_t0_]) × ([1 − Ht_tx_])/Ht_t__x_. These data were then used to calculate a UF-based correction factor for all patients (*n* = 40) with the formula: (1 − [UF/1000] × [1-average Ht/average UF]).

#### Correlations Between mDNA/APR and IDH

Because mDNA could not be detected at all and the APR disappeared after correction for hemoconcentration, analyses on associations between IDH episodes and either mDNA or the APR could not be performed.

## Results

### Patient Characteristics

Forty-five patients were included, 5 dropped out before randomization (Supplementary Figure S1). Baseline characteristics are summarized in Table 1. Of the patients 75% were male, mean age was 69.7 ± 13.5 years and median dialysis vintage was 3.0 (interquartile range: 1.0–5.8) years.

**Table 1:** Baseline characteristics of study participants

Demographics	*N* = 40
Sex (male), *n* (%)	30 (75)
Age (yrs)	69.7 ± 13.5
Ethnicity, *n* (%)	
Caucasian	28 (70)
African	10 (25)
Asian	2 (5)
Clinical characteristics	
BMI (kg/m^2^)	26.7 ± 4.2
Smoking status, *n* (%)	
Nonsmoker	14 (35)
Former smoker	18 (45)
Current smoker	8 (20)
Systolic blood pressure, predialysis (mm Hg)	145 ± 23
Diastolic blood pressure, predialysis (mm Hg)	81 ± 13
Residual kidney function^a^, *n* (%)	24 (60)
Residual kidney function (ml/min)^b^	1.9 (1.0–2.5)
Medical history	
Dialysis modality, *n* (%)	
HD	17 (42)
HDF	23 (58)
Dialysis vintage (yrs)	3.0 (1.0–5.8)
History of kidney transplantation, *n* (%)	3 (8)
Primary cause of ESKD, *n* (%)	
Glomerulonephritis	10 (25)
Renal vascular disease	9 (22)
Diabetic nephropathy	15 (38)
Cystic kidney disease	1 (2.5)
Other / Unknown	4 (10) / 1 (2.5)
Diabetes mellitus, *n* (%)	19 (48)
Hypertension, *n* (%)	28 (70)
History of CVD, *n* (%)	29 (73)
Medication, *n* (%)	
ACE-I/ARB	10 (25)
Beta-blockers	25 (63)
Calcium antagonists	10 (25)
Diuretics	11 (28)
ESA	32 (80)
Laboratory data	
Hemoglobin (mmol/l)	7.1 ± 0.7
Creatinine (μmol/l)	865 ± 229
Sodium (mmol/l)	138 ± 4
Potassium (mmol/l)	5.1 ± 0.6
Phosphate (mmol/l)	1.6 ± 0.5
Albumin (g/l)	38.6 ± 4.5
PTH (pmol/l)	28.2 (15.1–48.3)
Vascular access, *n* (%)	
AVF	32 (80)
CVC	4 (10)
Graft	4 (10)

Values are number (*n*) (%) for categorical variables; and mean ± SD or median (interquartile range) for continuous variables. Laboratory data are predialytic values.

ACE-I, angiotensin-converting enzyme inhibitor; ARB, angiotensin receptor blocker; AVF, arteriovenous fistula; BMI, body mass index; CVC, central venous catheter; CVD, cardiovascular disease; ESA, erythropoiesis-stimulating agent; ESKD, end-stage kidney disease; HD, hemodialysis; HDF, hemodiafiltration; PTH, parathyroid hormone.

a Residual diuresis >100 mL/24 h.

b In patients with diuresis >100 mL/24 h.

### Missing Data

Of the 40 patients who completed the study, 2 were not exposed to HDF but did complete S-HD and C-HD. Two other patients withdrew consent after completing 75% and 50% of the study. The inflammation markers of 1 patient were excluded because of a severe bacterial infection occurring after inclusion. No other serious adverse events occurred.

### Treatment Characteristics

Dialysis characteristics are shown in Supplementary Table S1. Mean blood flow in S-HD was 339 ± 33 ml/min; in C-HD 332 ± 41 ml/min; in LV-HDF 339 ± 36 ml/min; and in HV-HDF 347 ± 27 ml/min. Mean CV in LV-HDF was 15.1 ± 1.3 L per session and in HV-HDF 22.6 ± 1.1 L per session.

### IDH Incidence

During 458 dialysis sessions, 6939 blood pressure measurements were performed. The average number of IDH episodes was 0.68 per session in S-HD, 0.21 per session in C-HD, 0.51 per session in LV-HDF and 0.27 per session in HV-HDF.^13^

### mDNA

Because mDNA could not be detected in any blood sample (*n* = 88) of the first 11 patients, whereas all positive controls were confirmed by a positive test, this measurement was subsequently omitted.

### APR and Treatment Modality

**Figure 1:**
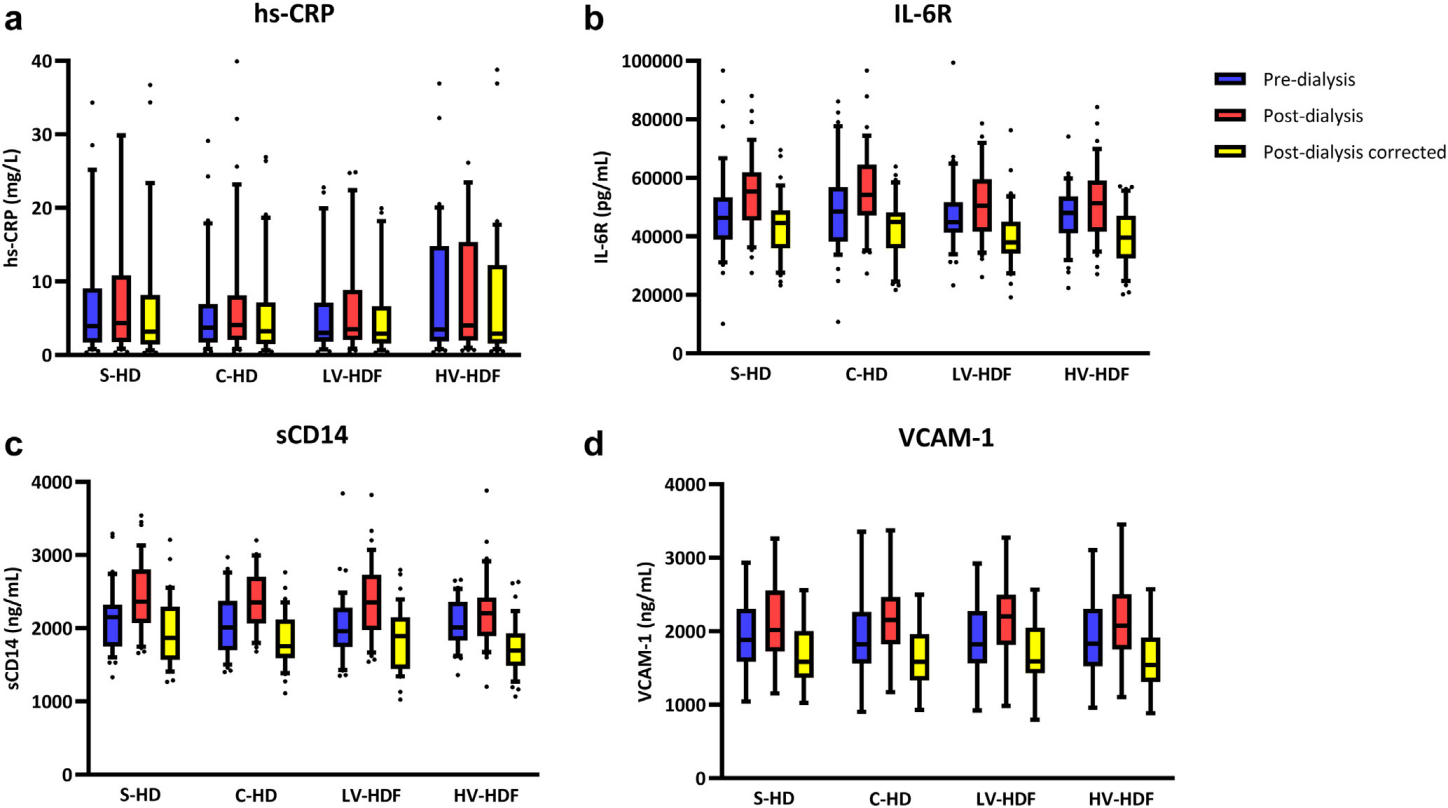
Predialysis and postdialysis concentration of APR markers (crude and corrected for hemoconcentration) among S-HD, C-HD, LV-HDF, and HV-HDF. (a) hs-CRP (mg/l). (b) IL-6R (pg/ml). (c) sCD14 (ng/ml). (d) VCAM-1 (ng/ml). Results are shown as boxplots; 25th and 75th percentile limit of the box; whiskers represent the maximum and minimum values. C-HD, cool hemodialysis; hs-CRP, high-sensitivity C-reactive protein; HV-HDF, high-volume hemodiafiltration; IL-6R, interleukin-6 receptor; LV-HDF, low-volume hemodiafiltration; sCD14, soluble CD14; S-HD, standard hemodialysis; VCAM-1, vascular-cell-adhesion molecule-1.

Predialytic values of hs-CRP, IL-6R, sCD14, and VCAM-1 did not differ between modalities (Figure 1, Table 2). Crude analyses showed that IL-6R, sCD14, and VCAM-1 increased (Table 2) in all modalities, without differences in the rates of change between modalities (*P*-values for interaction > 0.12). For hs-CRP, however, an increase was noted in C-HD and HV-HDF (*P* = 0.02 and *P* = 0.04, respectively), whereas changes were not observed in S-HD (*P* = 0.07) and in LV-HDF (*P* = 0.13).

**Table 2:** Change in APR markers: crude results

APR marker	Pre	Post	*P* pre^a^	Rate of change (95% CI)	*P*	*P* for interaction^b^
hs-CRP (mg/l)						
S-HD	3.9 (1.7–9.1)	4.3 (1.8–10.8)	0.43	1.3 (−0.1 to 2.7)	0.07	Ref.
C-HD	3.7 (1.7–7.0)	4.1 (2.1–8.1)		1.2 (0.2–2.2)	0.02	0.99
LV-HDF	3.0 (1.8–7.1)	3.5 (2.1–8.8)		1.8 (−0.6 to 4.1)	0.13	0.79
HV-HDF	3.5 (1.9–14.8)	4.0 (2.0–15.4)		1.6 (0.1–3.1)	0.04	0.86
IL-6R (ng/ml)						
S-HD	46.4 (38.9–53.4)	55.3 (45.5–61.9)	0.32	7.0 (1.7–12.4)	0.01	Ref.
C-HD	48.5 (38.2–56.8)	54.2 (47.2–64.5)		6.5 (0.9–12.1)	0.02	0.94
LV-HDF	44.9 (41.3–51.6)	50.5 (41.7–59.6)		3.9 (1.7–6.2)	0.001	0.29
HV-HDF	48.0 (41.0–53.7)	51.4 (41.7–59.1)		5.4 (2.4–8.4)	<0.001	0.56
sCD14 (ng/ml)						
S-HD	2150 (1750–2320)	2365 (2073–2800)	0.74	313.6 (178.9–448.3)	<0.001	Ref.
C-HD	2010 (1700–2370)	2350 (2065–2705)		347.5 (193.4–501.5)	<0.001	0.77
LV-HDF	1960 (1740–2280)	2350 (1975–2730)		346.2 (228.9–463.4)	<0.001	0.74
HV-HDF	2010 (1830–2360)	2205 (1890–2423)		195.5 (77.4–313.5)	0.002	0.23
VCAM-1 (ng/ml)						
S-HD	1880 (1580–2300)	2015 (1720–2553)	0.74	169.2 (103.7–234.8)	<0.001	Ref.
C-HD	1820 (1558–2263)	2150 (1820–2465)		223.1 (150.7–295.6)	<0.001	0.34
LV-HDF	1820 (1560–2270)	2200 (1810–2495)		260.7 (188.9–332.5)	<0.001	0.12
HV-HDF	1830 (1520–2300)	2075 (1750–2503)		165.6 (86.0–245.3)	<0.001	0.84

Predialysis and postdialysis concentration of APR markers expressed as median with interquartile range (*N* = 40). Rate of change (change in concentration in 4 h) shown as mean with 95% CI.

APR, acute phase response; C-HD, cool hemodialysis; CI, confidence interval; hs-CRP, high-sensitivity C-reactive protein; HV-HDF, high-volume hemodiafiltration; IL-6R, Interleukin-6 receptor; LV-HDF, low-volume hemodiafiltration; Post, postdialysis; Pre, predialysis; Ref, reference category; sCD14, soluble CD14; S-HD, standard hemodialysis; VCAM-1, vascular cell adhesion molecule-1.

a *P* value for difference in predialytic value between modalities.

b *P* value for difference in the rate of change of the respective modality in reference to S-HD.

### Correction for Hemoconcentration

After correction for hemoconcentration, the increase in APR markers were no longer present (Table 3). In contrast, a decrease was observed for sCD14 and VCAM-1 in all modalities (median decrease, −11.3% and −14.4%, respectively; *P* < 0.05), and for IL-6R in C-HD, LV-HDF and HV-HDF (median decrease, −13.4%; *P* < 0.05). For hs-CRP, a decrease was only shown in C-HD (median decrease, −13.5%; *P* = 0.004). The APR markers corrected for hemoconcentration in 11 patients with available Ht are shown in Supplementary Table S2. The changes corrected for hemoconcentration based on Ht (*n* = 11) were less pronounced than the changes corrected for hemoconcentration based on UF (*n* = 40) (Table 3).

**Table 3:** Change in APR markers: results corrected for hemoconcentration

APR marker	Pre	Post	*P* pre^a^	Rate of change (95% CI)	*P*	*P* for interaction^b^
hs-CRP (mg/l)						
S-HD	3.9 (1.7–9.1)	3.1 (1.4–8.1)	0.43	−0.6 (−1.2 to 0.0)	0.05	Ref.
C-HD	3.7 (1.7–7.0)	3.2 (1.5–7.2)		−0.4 (−0.7 to −0.1)	0.004	0.96
LV-HDF	3.0 (1.8–7.1)	2.9 (1.6–6.6)		−0.3 (−1.1 to 0.5)	0.46	0.90
HV-HDF	3.5 (1.9–14.8)	2.9 (1.6–12.2)		−0.6 (−1.2 to 0.0)	0.05	0.95
IL-6R (ng/ml)						
S-HD	46.4 (38.9–53.4)	44.5 (35.9–48.9)	0.32	−4.3 (−9.4 to 0.8)	0.10	Ref.
C-HD	48.5 (38.2–56.8)	44.9 (35.9–48.2)		−6.5 (−11.4 to −1.5)	0.01	0.51
LV-HDF	44.9 (41.3–51.6)	37.9 (34.1–45.1)		−7.4 (−9.3 to −5.4)	<0.001	0.25
HV-HDF	48.0 (41.0–53.7)	39.6 (32.5–47.1)		−6.3 (−8.9 to −3.6)	<0.001	0.44
sCD14 (ng/ml)						
S-HD	2150 (1750–2320)	1869 (1567–2293)	0.74	−185 (−317 to −54)	0.007	Ref.
C-HD	2010 (1700–2370)	1755 (1592–2119)		−236 (−352 to −119)	<0.001	0.60
LV-HDF	1960 (1740–2280)	1891 (1446–2149)		−171 (−256 to −85)	<0.001	0.96
HV-HDF	2010 (1830–2360)	1694 (1485–1932)		−311 (−401 to −222)	<0.001	0.14
VCAM-1 (ng/ml)						
S-HD	1880 (1580–2300)	1581 (1365–2000)	0.74	−265 (−331 to −199)	<0.001	Ref.
C-HD	1820 (1558–2263)	1582 (1329–1958)		−264 (−348 to −181)	<0.001	0.94
LV-HDF	1820 (1560–2270)	1588 (1430–2047)		−203 (−262 to −144)	<0.001	0.32
HV-HDF	1830 (1520–2300)	1541 (1308–1916)		−302 (−179 to −224)	<0.001	0.49

Predialysis and postdialysis concentration of APR markers expressed as median with interquartile range corrected for hemoconcentration (*N* = 40). Rate of change of APR markers (change in 4 h) shown as mean with 95% confidence interval.

APR, acute phase response; C-HD, cool hemodialysis; CI, confidence interval; hs-CRP, high-sensitivity C-reactive protein; HV-HDF, high-volume hemodiafiltration; IL-6R, Interleukin-6 receptor; LV-HDF, low-volume hemodiafiltration; Ref, reference category; sCD14, soluble CD14; S-HD, standard hemodialysis; VCAM-1, vascular cell adhesion molecule-1.

a *P* value of the difference in predialytic values between modalities.

b *P* value for the difference in the rate of change of the respective modality in reference to S-HD.

## Discussion

Mainly based on indirect evidence and retrospective analyses, IDH has been linked to organ hypoperfusion, tissue injury, and mortality.^29^^,^^35^ Because intestinal ischemia and subsequent mucosal damage may facilitate the translocation of bacteria from the bowel to the blood,^23^ potential differences between the 4 modalities in the appearance of circulating mDNA as well as the generation of an APR were primary objectives. Given that bacterial translocation may evoke a systemic inflammatory response, correlations between IDH and both mDNA and the APR were the secondary aims of this study. In fact, 3 main conclusions can be drawn. First, mDNA could not be detected at all, despite the use of a high-sensitive state-of-the-art technique. Second, a comparable APR was observed in all modalities which, however, disappeared or even reversed after correction for hemoconcentration. Third, because the appearance of mDNA in blood is considered an indirect proxy for gut mucosal damage, our negative results challenge the idea that extracorporeal replacement therapy-induced intestinal ischemia promotes bacterial translocation.

Therefore, it is all the more remarkable that circulating bacterial DNA was demonstrated in previous studies, both in patients who were treated with peritoneal dialysis,^36^ HD,^37^^,^^38^ and patients with chronic kidney disease who are not yet on dialysis.^39^ So, why are our results diametrically opposed to these investigations, which showed not only mDNA in blood, but even associations with inflammation^36^^,^^38^^,^^40^^,^^41^ and cardiovascular disease?^22^^,^^23^ The question about whether circulating DNA is present is difficult and cannot conclusively be answered. The detection and isolation of bacterial products, such as lipopolysaccharide and other bacterial exotoxins in the past century^42^ triggered Schindler to look for the presence of mDNA or its short fragments in dialysate.^43^ After publishing conformational results,^43^ other groups investigated mDNA in the blood of patients on HD.^36^, ^37^, ^38^, ^39^, ^40^, ^41^^,^^44^, ^45^, ^46^ However, in these studies, mDNA was extracted from only 200 μl of full blood, followed by amplification of long mDNA fragments (deriving from 16S rDNA) and extraction of amplicons from agarose gel, followed by sequencing of fragments. More recently, however, it has become clear that this approach is not suited for the detection of intact mDNA. Moreover, the recent surge of interest in eubacterial detection by next generation sequencing has unraveled the high risk of contamination, either from well-to-well^47^^,^^48^ or by contamination of reagents,^49^, ^50^, ^51^ especially in low-biomass samples (with a low expected bacterial load).^49^^,^^51^, ^52^, ^53^ The protocol employed in this study, combining the Polaris extraction method with the molecular culture assay is an example of a new generation protocol aiming at maximizing sensitivity, while minimizing contamination in low biomass samples. Interestingly, our negative results are supported by a recent large analysis, in which a comparable high-sensitive technique to detect bacterial ribosomal DNA in blood was applied.^54^ Contrary to studies, which suggested a common blood microbiome in healthy individuals,^55^ mDNA was absent after implementing rigorous contamination controls and sequencing artifact checks. In fact, these findings suggest that the positive findings of previous studies resulted most likely from technical imperfections and/or contamination. Therefore, our results strongly challenge the idea that dialysis allows the translocation of intact bacteria from the gut to the blood.

With respect to the APR, our data seems rather puzzling at first sight. Whereas hs-CRP, IL-6R, sCD14, and VCAM-1 increased in the crude analysis, no change or even decreasing values were observed after correction for hemoconcentration. The latter findings contrast with many previous studies, generally reporting only uncorrected data^56^, ^57^, ^58^, ^59^ and thus incorrectly suggesting that HD treatment itself provokes an acute inflammatory response. However, from the few investigations that took hemoconcentration into account, it appeared that no APR occurred,^60^, ^61^, ^62^ which is in line with our findings. Furthermore, it should be realized that production and release of CRP by the liver takes 8 to 10 hours.^63^ Accordingly, we previously showed that CRP levels did not rise during HD with cuprophane dialyzers, but indeed in samples which were taken 20 hours later.^64^ Changes were not found when polyethersulfone or polysulfone membranes were used.^64^ Therefore, hemoconcentration rather than acute production seems to explain the “APR” in previous uncorrected studies. Moreover, the current widespread use of biocompatible dialyzers and the application of ultrapure dialysis fluid may also contribute to the absence of an APR.^64^^,^^65^ Still, it remains unclear why APR markers even declined in our investigation. At this point, it should be mentioned that dialyzer membranes, depending on their structure and charge, can bind various substances, including mediators of inflammation.^66^^,^^67^ Therefore, an increase will only be observed when release prevails over adsorption. Because the APR markers used in this study are too large to cross the dialyzer membrane, adsorption may have occurred. Future dialyzer elution studies may be helpful to answer this question.^68^

To the best of our knowledge, this is the first study analyzing both mDNA and the APR during 4 frequently applied extracorporeal replacement therapies. The largest strength is its randomized cross-over design, which prevents interindividual differences, such as comorbidity and medication, from influencing the results. A second strength is the quantitative assessment of bacterial DNA by the high-sensitive and sophisticated 16S-23S IS-pro technique after DNA isolation with a protocol optimized for detection of mDNA in large blood volumes (5.0 ml).^31^^,^^32^ A limitation, which appears especially relevant afterward, is the lack of simultaneous lipopolysaccharide determinations. Because measuring endotoxins in blood and dialysate is relatively unreliable,^69^ in this study only mDNA from intact bacteria was assessed. Nonetheless, because translocation of gut-derived bacterial products, such as lipopolysaccharide or bacterial fragments, cannot be completely excluded, future studies should pay attention to this option. Besides, considering that this study only concerns processes in the intradialytic acute phase, we lack interdialytic or long-term data. Furthermore, because Ht-values were only available in 11 patients, we computed a correction-factor based on the mean Ht change/1000 ml UF. Because the decrease in APR markers in these 11 patients was less pronounced after correction for Ht than with the formula (Supplementary Table S2), our calculations may overestimate the true effect.

In summary, intradialytic bacterial translocation could not be demonstrated despite the use of a highly sensitive state-of-the-art technique for measuring mDNA in blood. Therefore, our findings challenge the view that the superior survival in HV-HDF is due to less IDH-induced tissue damage, at least as far as the bowel is concerned. Whether other vital organs, including the heart, benefit from the low IDH-incidence in this modality remains to be established.

## Disclosure

MJN, MPCG, and PAR report unrestricted grant support, paid to the institution, from Niercentrum aan de Amstel, Elyse Klinieken, the Netherlands and B. Braun Avitum AG, Melsungen, Germany. AEB has proprietary rights on the IS-pro technology and is a cofounder of the Amsterdam UMC location Vrije Universiteit spin-off company IS-diagnostics. All the other authors declared no competing interests.

## Supplementary Material

Supplementary File (JPG and PDF) **Figure S1.** Study flowchart. **Table S1.** Dialysis characteristics. **Table S2.** Change in APR markers: results corrected for hemoconcentration (*n* = 11). CONSORT 2010 checklist of information when reporting a randomized trial. CONSORT 2010 flow diagram.

## Footnotes

Supplementary File (JPG and PDF)
**Figure S1.** Study flowchart. **Table S1.** Dialysis characteristics. **Table S2.** Change in APR markers: results corrected for hemoconcentration (n = 11). CONSORT 2010 checklist of information when reporting a randomized trial. CONSORT 2010 flow diagram.
